# Sex-based survival disparities in patients with stage I–III non-small cell lung cancer vary by treatment modality

**DOI:** 10.3389/fonc.2026.1868017

**Published:** 2026-09-18

**Authors:** Guus R.M. van den Heuvel, Bas L.R. Mandos, Hans J.M. Smit, Olga C.J. Schuurbiers, Erik H. J. G. Aarntzen, Iris Walraven, Francesco Ciompi, Michel M. van den Heuvel

**Affiliations:** 1 Department of Pulmonology, Radboud University Medical Center, Nijmegen, Netherlands; 2 Department of Pulmonology, Rijnstate, Arnhem, Netherlands; 3 Department of Medical Imaging, Radboud University Medical Center, Nijmegen, Netherlands; 4 Department of Medical Imaging, Groningen University Medical Center, Groningen, Netherlands; 5 Department of Intelligent Quality (IQ) Health, Radboud University Medical Center, Nijmegen, Netherlands; 6 Department of Computational Pathology, Radboud University Medical Center, Nijmegen, Netherlands; 7 Department of Pulmonology, University Medical Center, Utrecht, Netherlands

**Keywords:** NSCLC, sex, stage I-III, survival, treatment modalities

## Abstract

**Introduction:**

Female sex is a well-established prognostic factor in non-small cell lung cancer (NSCLC). Most prior studies are restricted to single-treatment cohorts and seldom include best supportive care (BSC), limiting the evaluation of sex-based median overall survival across the full treatment spectrum, BSC included.

**Methods:**

We conducted a retrospective study of patients diagnosed with clinical stage I–III NSCLC treated with curative intent (surgery, radiotherapy, or chemoradiation) or primary BSC. Survival rate was analyzed using log-rank tests and multivariable Cox regression to adjust for confounding prognostic factors.

**Results:**

In the total curative treatment cohort (n=1634), median overall survival (mOS) was 74.5 and 44.6 months in female and male patients, respectively [hazard ratio (HR) 0.60, P<0.001]. However, in the BSC cohort (n=234), mOS was similar between female (5.9 months) and male (5.8 months) patients (HR 0.93, P = 0.60). After stratification, mOS was not reached (females) versus 66.4 months (males) after surgery, (HR 0.58, P<0.001); 46.2 months (females) versus 33.0 months (males) after radiotherapy (HR 0.64, P<0.001); and 39.3 months (females) versus 26.9 months (males) after chemoradiation (HR 0.63, P<0.001). After multivariable adjustment, female survival benefit remained significant after curative-intent treatment overall (HR 0.72, P<0.001), surgery (HR 0.69, P = 0.003), and chemoradiation (HR 0.72, P = 0.02), but not after radiotherapy (HR 0.80, P = 0.12).

**Conclusions:**

Female survival advantage in stage I–III NSCLC is treatment-dependent and absent in the BSC cohort. Prospective studies with more detailed clinical, biological, and sociodemographic data are needed to determine whether sex independently affects survival or reflects other prognostic factors.

## Introduction

Female sex is consistently associated with a favorable prognosis in non-small cell lung cancer (NSCLC) (1, 2). Most prior studies assessed sex-based survival differences at an aggregate level without accounting for treatment modality, thereby risking confounding by indication and obscuring potential heterogeneity in treatment-specific outcomes. Treatment-specific comparisons in patients with stage I–III NSCLC have been reported, particularly in surgical and radiotherapy cohorts (3–6). However, most studies evaluated sex-based survival differences within single-treatment cohorts—most commonly populations with resected NSCLC— thereby limiting generalizability and precluding assessment of whether observed disparities are consistent across therapeutic contexts. While such designs reduce heterogeneity within a given modality, they do not address whether sex-based differences are treatment-independent or instead reflect interactions with specific interventions. Consequently, findings from single-modality cohorts may overestimate or misattribute prognostic effects by failing to account for treatment selection processes and modality-specific dynamics.

Curative-intent therapy comprising surgery, radiotherapy, or definitive chemoradiation remains the cornerstone of treatment for patients with clinical stage I–III NSCLC (7, 8). Patients who are not considered candidates for active anticancer treatment and instead receive best supportive care (BSC) represent a distinct population. In the Netherlands, 9%, 15%, and 25% of patients with clinical stages I, II, and III NSCLC, respectively, received BSC as primary treatment (9). A BSC cohort typically harbors patients with a high comorbidity burden, poor performance status, and other barriers to curative treatment. It is not known whether a female survival advantage persists in this population. In this context, patients receiving BSC represent a clinically and methodologically informative subgroup. As these patients do not undergo tumor-directed treatment, the influence of treatment-related selection and intervention is minimized. The BSC population can therefore serve as a quasi-reference group to assess whether sex-based survival differences persist in the relative absence of treatment effects and associated confounding.

In this study, we evaluated whether sex-based differences in survival among patients with stage I–III NSCLC vary according to initial treatment approach including primary BSC. We hypothesized that the observed survival advantage in female patients is at least partly attributable to confounding related to treatment selection and patient fitness, and that this effect would be attenuated or absent in the BSC population.

## Methods

### Objectives

We investigated whether the survival advantage associated with female sex in patients with stage I–III NSCLC varies according to primary treatment modality. First, we sought to evaluate whether female sex is independently associated with improved overall survival among patients within the total curative-intent treatment cohort and the primary BSC cohort. Second, we performed a stratified analysis to assess whether this association varies across treatment modalities within the curative-intent cohort, including surgery, radiotherapy, and chemoradiation. The radiotherapy cohort was stratified into a stereotactic ablative and conventional radiotherapy subcohort; the chemoradiation cohort was stratified according to the administration of adjuvant durvalumab. Finally, we conducted a comparison of disease-free survival (DFS) across the curative-treatment subcohorts.

### Study design

Patients with clinical stage I–III NSCLC diagnosed between 2015 and 2019 were included. Data collection was performed until December 2023. A minimum follow-up duration of 4 years was pursued. Patients were diagnosed in hospitals participating in the Lung Cancer Network^1^, a regional network in the Eastern part of the Netherlands. Patients received either curative-intent treatment (surgery, radiotherapy, or chemoradiation) or primary BSC. Patients participating in a clinical interventional study and whose stage was unknown were excluded. Overall survival was defined as the time from diagnosis to death from any cause. DFS was defined as the time from diagnosis until recurrence or death from any cause. Surviving patients were censored at their latest follow-up. This retrospective study was approved by the Dutch Clinical Research Foundation and was carried out in accordance with ethical principles in the Declaration of Helsinki. The study was not subject to the Medical Research Involving Human Subjects Act.

### Statistical analysis

Distribution patterns were evaluated using Chi-square tests for categorical variables and Student’s t-tests for continuous variables. OS and DFS were analyzed using Kaplan–Meier curves and compared with log-rank tests. The Cox proportional hazard regression model was used to determine hazard ratios (HRs). Hazard ratios were adjusted (aHR) for relevant confounders: age, Eastern Cooperative Oncology Group Performance Score (ECOG PS), smoking history, Charlson co-morbidity index (CCI), histopathology (including “imaging only”), clinical stage, and treatment strategy. Statistical analyses were performed using SPSS version 29.0 and GraphPad version 10.2.2. Forest plots were built in RStudio version 4.4.1 with base, graphic, and forestplot packages. P-values were calculated for HRs using two-sided tests. Values of ≤0.05 were considered statistically significant.

## Results

### Baseline characteristics

In total, 1,918 patients were evaluated for eligibility. After the exclusion of study participants and patients with unknown stage, 858 female patients and 1,010 male patients were included (N = 1,868 in total) (**Supplementary Figure 1**). In the total curative treatment study cohort, 783 (48%) female patients and 851 (52%) male patients were included (**Supplementary Table 1**). Age at diagnosis, smoking history, histopathology, clinical stage, and the CCI were imbalanced in favor of the female patient population. In the primary BSC study cohort, 75 female patients (32%) and 159 male patients (68%) were included. Clinical and tumor characteristics showed a balanced distribution in the primary BSC cohort between female and male patients (**Supplementary Table 1**).

### Sex-based survival in the curative treatment and BSC cohorts

In the total curative treatment cohort (n=1,634), median overall survival (mOS) was 74.5 months in female patients versus 44.6 months in male patients (HR 0.60, 95% CI 0.53 to 0.69; P<0.001) (**Figure 1A**). This finding was consistent after adjustment for confounders (aHR 0.72, 95% CI 0.62 to 0.84; P<0.001) (**Figure 1B**).

**Figure 1:**
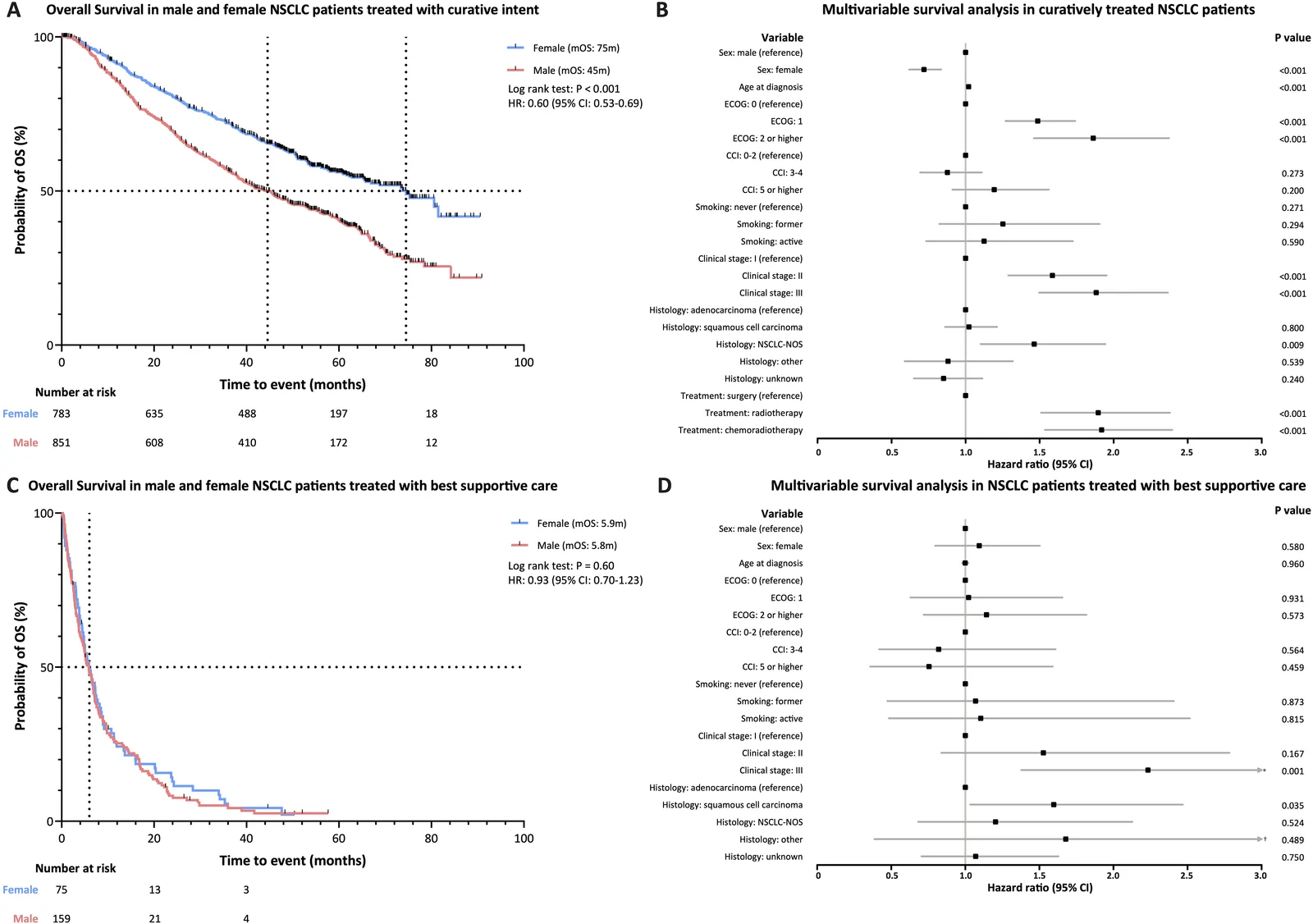
Sex-based survival rates and covariates on the hazard of an event in patients with stage I–III NSCLC treated with either curative intent or primary BSC in a multivariable Cox regression model. **(A)** Overall survival in female versus male patients in the curative treatment cohort (N = 1,634). **(B)** Multivariable Cox regression model in the curative treatment cohort (N = 1,529). **(C)** Overall survival in female versus male patients in the primary BSC cohort (N = 234). **(D)** Multivariable Cox regression model in the primary BSC cohort (N = 199). NSCLC NOS, non-small cell lung cancer not otherwise specified; BSC, best supportive care; CRT, chemoradiotherapy; RT, radiotherapy; and ECOG PS, Eastern Cooperative Oncology Group performance score. * 95% CI upper limit of 3.62. * 95% CI upper limit of 7.27.

In the primary BSC cohort (n=234), mOS was 5.9 months in female versus 5.8 months in male patients (HR 0.93, 95% CI 0.70 to 1.23; P = 0.60) (**Figure 1C**). No significant difference was observed after adjustment for prognostic factors (aHR 1.09, 95% CI 0.80 to 1.50; P = 0.58) (**Figure 1D**).

### Surgery cohort

Demographics of the surgery cohort (n=876) are summarized in **Supplementary Table 2**. mOS was not reached in female patients versus 66.4 months in male patients (HR 0.58, 95% CI 0.46 to 0.72; P<0.001) (**Figure 2A**). This benefit persisted after adjustment for confounders (aHR 0.69, 95% CI 0.54 to 0.88; P = 0.003) (**Figure 2B**). Median DFS was not reached in females versus 55.0 months in males (HR 0.68, 95% CI 0.56 to 0.83; P<0.001). This gap was maintained after adjustment (aHR 0.78, 95% CI 0.63 to 0.96; P = 0.02).

**Figure 2:**
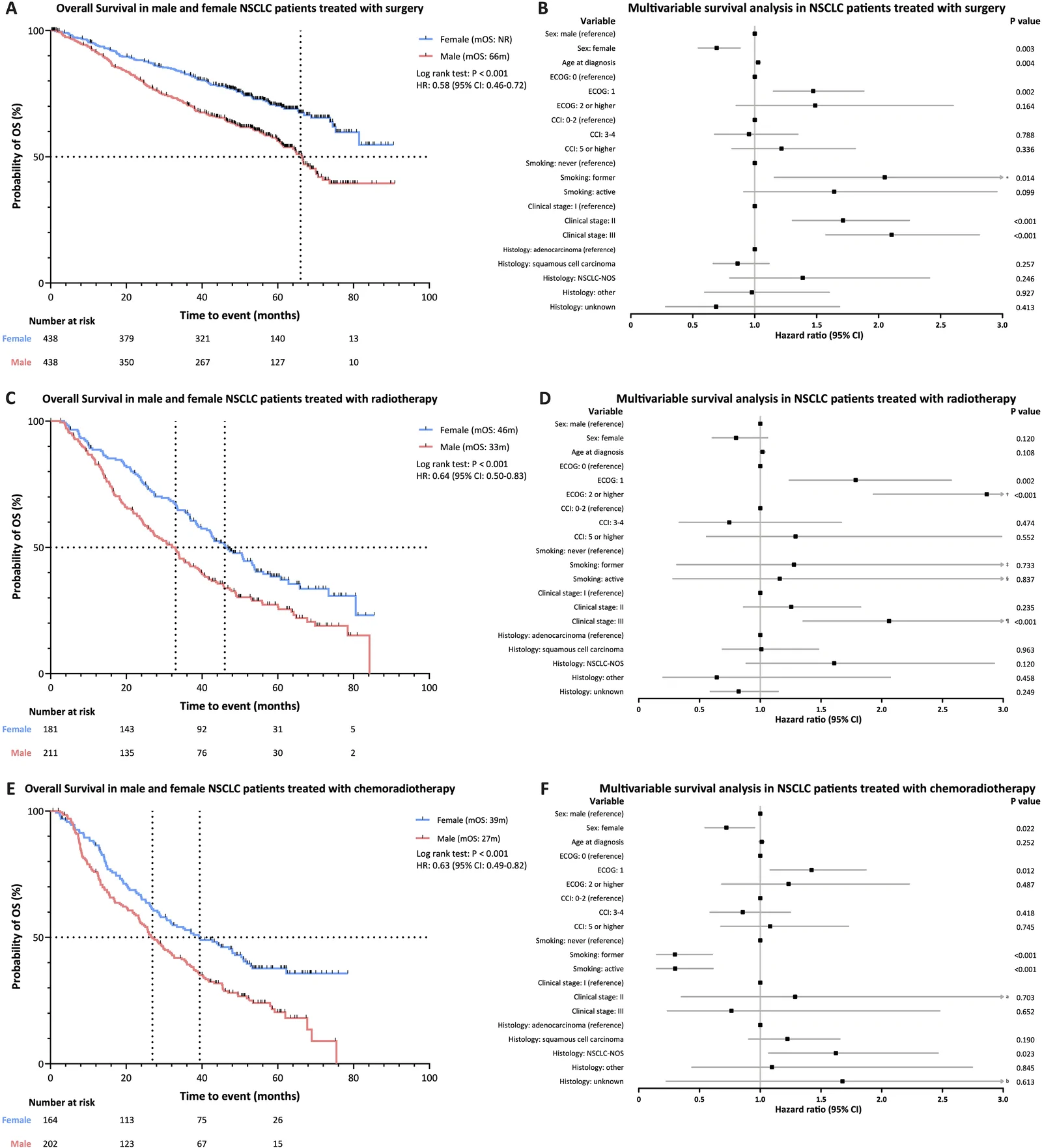
Sex-based survival rates and covariates on the hazard of an event in patients with stage I–III NSCLC stratified to curative-intent treatment strategy. Female-to-male probability of OS stratified to surgical treatment **(A)**, radiotherapy **(B)**, and chemoradiation **(C)**. NSCLC, non-small cell lung cancer and BSC, best supportive care. **(A)** Overall survival in female versus male patients in the surgery cohort (N = 876). **(B)** Multivariable Cox regression model in the surgery cohort (N = 832). **(C)** Overall survival in female versus male patients in the radiotherapy cohort (N = 392). **(D)** Multivariable Cox regression model in the radiotherapy cohort (N = 347). **(E)** Overall survival in female versus male patients in the chemoradiation cohort (N = 366). **(F)** Multivariable Cox regression model in the chemoradiation cohort (N = 350). *95% CI upper limit of 3.63. † 95% CI upper limit of 4.25. ‡ 95% CI upper limit of 5.21. § 95% CI upper limit of 4.80. ¶ 95% CI upper limit of 3.14. a 95% CI upper limit of 4.72. b 95% CI upper limit of 12.43.

### Radiotherapy cohort

Demographics of the radiotherapy cohort (n=392) are summarized in **Supplementary Table 3**. mOS was 46.2 months in female patients versus 33.0 months in male patients (HR 0.64, 95% CI 0.50 to 0.83; P<0.001) (**Figure 2C**). This benefit did not persist after adjustment for confounders (aHR 0.80, 95% CI 0.61 to 1.06; P = 0.12) (**Figure 2D**). DFS was 42.6 months in females versus 24.6 months in males (HR 0.63, 95% CI 0.49 to 0.80; P<0.001). This disparity was prolonged after adjustment (aHR 0.76, 95% CI 0.58 to 0.99; P = 0.04).

In the stereoablative subcohort (n=322), mOS was 50.5 months in female patients versus 36.5 months in male patients (HR 0.66, 95% CI 0.50 to 0.87; P = 0.004) (**Supplementary Figure 2A**). This benefit did not hold after adjustment for confounders (aHR 0.81, 95% CI 0.59 to 1.11; P = 0.19). In the conventional radiotherapy subcohort (n=61), mOS was 21.3 versus 19.0 months in female versus male patients (HR 0.75, 95% CI 0.42 to 1.33; P = 0.32) (**Supplementary Figure 2B**). No significant benefit favoring female patients was observed after adjustment for confounders (aHR 0.93, 95% CI 0.44 to 1.97; P = 0.85).

### Chemoradiation cohort

Demographics of the chemoradiation cohort (n=366) are summarized in **Supplementary Table 4**. MOS was 39.3 months in female patients versus 26.9 months in male patients (HR 0.63, 95% CI 0.49 to 0.82; P<0.001) (**Figure 2E**). This benefit remained after adjustment for confounders (**Figure 2F**) (aHR 0.72, 95% CI 0.55 to 0.95; P = 0.02). DFS was 19.4 months in females versus 15.7 months in males (HR.80, 95% CI 0.63 to 1.006). After adjustment, no significant gap was observed (aHR 0.83, 95% CI 0.64 to 1.07; P = 0.15).

In the subcohort of patients treated with adjuvant durvalumab after chemoradiation (n=117), mOS was not reached in female patients versus 45.8 months in male patients (HR 0.69, 95% CI 0.40 to 1.18) (**Supplementary Figure 3A**). After adjustment for confounders, again, no significant mOS disparity was observed (aHR 0.64, 95% CI 0.35 to 1.17; P = 0.15). In the chemoradiation subcohort without adjuvant durvalumab (n=249), mOS was 28.7 months in female patients versus 22.5 months in male patients (HR 0.64, 95% CI 0.48 to 0.86) (**Supplementary Figure 3B**). A survival benefit was also demonstrated after adjustment for confounders (aHR 0.70, 95% CI 0.50 to 0.97; P = 0.03).

## Discussion

In this large, multicenter retrospective cohort of patients with stage I–III NSCLC, female sex was associated with a significantly improved overall survival within the curative-intent treatment population. This association persisted after multivariable adjustment and was most pronounced in the surgery and chemoradiation cohorts. In contrast, no survival difference between sexes was observed in the primary best supportive care (BSC) cohort. While these findings may suggest a sex-related survival advantage in patients eligible for curative treatment, the observed disparities are likely influenced, at least in part, by residual confounders and should therefore be interpreted with caution.

A key observation of this study is the imbalance in baseline characteristics (**Table 1**) between female and male patients. Women generally presented with favorable clinical profiles, based on age at diagnosis, smoking history, tumor histopathology, clinical stage, and comorbidity burden. These imbalances suggest that female patients entered curative treatment pathways with advantageous prognostic features, which may partly contribute to observed survival disparities. In contrast, within the primary BSC cohort, baseline clinical and tumor-related characteristics were well balanced between sexes. This balance reduces the likelihood that baseline disparities confounded survival comparisons in the non-curative setting. In a large Japanese registry study including patients with resectable NSCLC, a higher incidence of stage IA and adenocarcinoma subtype was assumed to account for a better prognosis in women (3). Moreover, in an Australian prospective study, sex-related survival disparities in lung cancer patients were largely attributed to prognostic factors, such as tobacco smoking, lifestyle, and treatment modality. After adjusting for these features, no significant difference in OS across female and male patients was observed (4). Consequently, the apparent survival benefit in female patients may, to some extent, reflect a more favorable baseline risk profile rather than a true independent effect of sex.

**Table 1:** Baseline characteristics of patients with stage I-III NSCLC treated with either curative-intent therapy or primary BSC.

Demographics	Curative treatment cohort			Primary BSC cohort		
Population	Female	Male	P-value	Female	Male	P-value
	n=795	n=866		n=75	n=159	
Age at diagnosis—years			<0.001			0.50
Mean ± SD	66.2 ± 9.6	69.3 ± 9.3		75.5 ± 9.3	76.4 ± 9.3	
Median(range)	67 (32-93)	70 (18-89)		76 (49-93)	79 (48-98)	
ECOG PS at diagnosis—no (%)			0.37			0.93
0	428 (54)	440 (51)		9 (12)	17 (11)	
1	267 (33)	319 (37)		23 (31)	47 (30)	
>1	79 (10)	86 (10)		37 (49)	82 (51)	
Unknown	21 (3)	21 (2)		6 (8)	13 (8)	
Smoking status—no (%)			<0.001			0.11
Current	334 (42)	296 (34)		30 (40)	65 (41)	
Former	381 (48)	510 (59)		36 (48)	77 (48)	
Never	56 (7)	32 (4)		5 (7)	2 (1)	
Unknown	24 (3)	28 (3)		4 (5)	15 (9)	
Year of diagnosis—no (%)			0.18			0.33
≤2016	47 (6)	74 (9)		5 (6)	11 (7)	
2017	238 (30)	263 (30)		20 (27)	55 (35)	
2018	252 (32)	252 (29)		20 (27)	48 (30)	
2019	258 (32)	277 (32)		30 (40)	45 (28)	
Type of diagnosis—no (%)			0.85			0.48
Pathologically proven	702 (88)	762 (88)		48 (64)	94 (59)	
Imaging only	93 (12)	104 (12)		27 (36)	65 (41)	
Histopathology if known—no (%)			<0.001			0.10
Adenocarcinoma	415 (59)	333 (44)		22 (46)	27 (29)	
Squamous cell carcinoma	187 (27)	331 (43)		16 (33)	52 (55)	
NSCLC NOS	43 (6)	60 (8)		8 (17)	12 (13)	
Other	57 (8)	38 (5)		2 (4)	3 (3)	
Clinical stage at baseline—no (%)			<0.001			0.54
I	409 (52)	361 (42)		10 (13)	17 (11)	
II	147 (18)	172 (20)		13 (17)	21 (13)	
III	239 (30)	333 (38)		52 (70)	121 (76)	
Main treatment strategy—no (%)			0.34			N/A
Surgery	438 (55)	438 (51)				
Radiotherapy	181 (23)	211 (24)				
Chemoradiation	176 (22)	217 (25)				
Charlson comorbidity index—no(%)			<0.001			0.97
Mild (0-2)	223 (28)	156 (18)		6 (8)	13 (8)	
Moderate (3-4)	306 (39)	315 (36)		22 (29)	44 (28)	
Severe (≥5)	266 (33)	395 (46)		47 (63)	102 (64)	

In patients who underwent surgical resection, female sex was associated with superior survival. Importantly, this benefit persisted after adjustment for relevant clinical and tumor-related confounders, suggesting an independent association. These findings were consistent with results from a Swedish nationwide study, which demonstrated a lower risk of death among women compared with men, even after adjustment for prognostic confounders persistent after adjustment for prognostic confounders (5). In a large Chinese retrospective cohort (2024), female patients with NSCLC demonstrated lower incidence of complications and a shorter length of stay after resection (6). These findings indicate that sex-related biological or treatment-response differences may be particularly relevant in surgically treated populations.

In the radiotherapy cohort, female patients initially appeared to have improved survival outcomes. However, this apparent advantage did not remain statistically significant after adjustment for confounding variables. Similar patterns were observed in both stereotactic and conventional radiotherapy subgroups. On the contrary, in a retrospective cohort study of McGovern et al. (2009), including 831 patients with stage I–III NSCLC treated with radiotherapy, female sex was associated with better OS (10). This survival benefit persisted after multivariable analysis including adjustment for age, smoking status, clinical stage, and Karnofsky score. Attenuation of a female survival benefit after adjustment suggests that the crude association was largely mediated by disparities in tumor biology and clinical characteristics rather than sex itself. Biological mechanisms, including differences in histology, molecular alterations, and hormonal influences may confer a survival advantage in women but are partly captured by adjusted covariates. Alternatively, residual confounding or overadjustment may have contributed to our findings.

In the chemoradiation cohort, female patients demonstrated a significantly longer overall survival compared with male patients, an association that remained significant after adjustment for relevant confounders. In a Japanese comparative study (2009), no sex difference was shown after comparison of female versus male patients with NSCLC treated with chemoradiation (11). However, lack of a survival disparity may reflect insufficient statistical power, given the small number of female patients in the subgroup (n=56).

Several factors could contribute to sex-related survival disparities. First, evidence suggests that biological differences in immune function between sexes may contribute to the observed survival advantage among female patients. For instance, the tumor microenvironment is reported to be more enriched for effector CD4^+^ and CD8^+^ T cells in female patients with NSCLC (12). Consequently, a more robust anticancer response may be generated by treatment modalities that have immune-mediated mechanisms of action, such as chemotherapy and radiotherapy (13, 14). In addition, potential roles for sex steroids, Follicle-Stimulating Hormone (FSH), Luteinizing Hormone (LH) and activin/inhibin were discovered in order to explain different immune responses to programmed-death (PD)-1 therapies (15, 16). Second, female patients tend to seek medical attention earlier. Sex-based lung cancer care-seeking and utilization behaviors may be significant contributors to lung cancer-related morbidity and mortality (17). This phenomenon may not have the same impact in a primary BSC setting, where medical interventions are limited to symptom management. Finally, nutritional status, molecular phenotyping, and tumor differentiation are known prognostic factors in the context of localized NSCLC and may further contribute to observed sex-related survival disparities (18).

There are some limitations. First, for most variables, patients with missing values were excluded from multivariable Cox regression analyses. For histopathology, patients without tissue confirmation were retained as a separate category, as it reflects a clinically relevant subgroup rather than true missing data. Second, this study lacks data on several known prognostic factors in NSCLC. For example, nutritional status, molecular phenotyping, and tumor differentiation were not available in our dataset. Finally, patients without documented clinical stage were excluded, which may have introduced selection bias. In our study population, 30 patients had unknown stage, of whom 27 patients (90%) had primary BSC.

## Conclusion

While female sex was associated with improved overall survival in patients undergoing curative-intent treatment for stage I–III NSCLC, this apparent advantage is likely multifactorial and potentially driven by baseline imbalances and residual confounding. The absence of a survival difference in the BSC cohort supports this interpretation. Future prospective studies with more granular clinical, biological, and sociodemographic data are warranted to clarify whether sex independently influences survival outcomes or primarily acts as a surrogate for other prognostic factors.

## Data Availability

The raw data supporting the conclusions of this article will be made available by the authors, without undue reservation.

## Footnotes

1 Lung Cancer Network with participating centers Bernhoven Uden, Canisius Wilhelmina Hospital Nijmegen, Elkerliek Helmond, Gelderse Vallei Ede, Jeroen Bosch Hospital’s- Hertogenbosch, Pantein Boxmeer, Radboudumc Nijmegen, Rijnstate Arnhem, Slingeland Hospital Doetinchem
