# Supplementary material for: Sex-based survival disparities in patients with stage I–III non-small cell lung cancer vary by treatment modality
Source: Front Oncol. 2026 Sep 18;16:1868017. doi: 10.3389/fonc.2026.1868017 (PMC13630633; doi:10.3389/fonc.2026.1868017)
Supplement: Supplementary file 1 [file Table1.docx]

| **Demographics** | **Curative treatment cohort** |  |  | **Primary BSC cohort** |  |  |
| --- | --- | --- | --- | --- | --- | --- |
| **Population** | **Female** | **Male** | **P-value** | **Female** | **Male** | **P-value** |
|  | n=783 | n=851 |  | n=75 | n=159 |  |
| **Age at diagnosis—years** |  |  | **<0.001** |  |  | 0.50 |
| Mean±SD | 66.2 ± 9.6 | 69.3 ± 9.3 |  | 75.5 ± 9.3 | 76.4 ± 9.3 |  |
| Median(range) | 67 (32-93) | 70 (18-89) |  | 76 (49-93) | 79 (48-98) |  |
| **ECOG PS at diagnosis—no(%)** |  |  | 0.32 |  |  | 0.93 |
| 0 | 426 (55) | 437 (51) |  | 9 (12) | 17 (11) |  |
| 1 | 260 (33) | 313 (37) |  | 23 (31) | 47 (30) |  |
| >1 | 78 (10) | 81 (10) |  | 37 (49) | 82 (51) |  |
| Unknown | 19 (2) | 20 (2) |  | 6 (8) | 13 (8) |  |
| **Smoking status—no(%)** |  |  | **<0.001** |  |  | 0.11 |
| Current | 334 (43) | 292 (34) |  | 30 (40) | 65 (41) |  |
| Former | 375 (48) | 499 (59) |  | 36 (48) | 77 (48) |  |
| Never | 56 (7) | 32 (4) |  | 5 (7) | 2 (1) |  |
| Unknown | 18 (2) | 28 (3) |  | 4 (5) | 15 (9) |  |
| **Year of diagnosis—no(%)** |  |  | 0.16 |  |  | 0.33 |
| ≤2016 | 46 (6) | 74 (9) |  | 5 (6) | 11 (7) |  |
| 2017 | 234 (30) | 255 (30) |  | 20 (27) | 55 (35) |  |
| 2018 | 248 (32) | 250 (29) |  | 20 (27) | 48 (30) |  |
| 2019 | 255 (32) | 272 (32) |  | 30 (40) | 45 (28) |  |
| **Type of diagnosis—no(%)** |  |  | 0.83 |  |  | 0.48 |
| Pathologically proven | 690 (88) | 747 (88) |  | 48 (64) | 94 (59) |  |
| Imaging only | 93 (12) | 104 (12) |  | 27 (36) | 65 (41) |  |
| **Histopathology if known—no(%)** |  |  | **<0.001** |  |  | 0.10 |
| Adenocarcinoma | 409 (59) | 331 (44) |  | 22 (46) | 27 (29) |  |
| Squamous cell carcinoma | 183 (27) | 323 (43) |  | 16 (33) | 52 (55) |  |
| NSCLC NOS | 43 (6) | 56 (8) |  | 8 (17) | 12 (13) |  |
| Other | 55 (8) | 37 (5) |  | 2 (4) | 3 (3) |  |
| **Clinical stage at baseline—no(%)** |  |  | **<0.001** |  |  | 0.54 |
| I | 409 (52) | 361 (42) |  | 10 (13) | 17 (11) |  |
| II | 146 (19) | 171 (20) |  | 13 (17) | 21 (13) |  |
| III | 228 (29) | 319 (38) |  | 52 (70) | 121 (76) |  |
| **Main treatment strategy—no(%)** |  |  | 0.18 |  |  | N/A |
| Surgery | 438 (56) | 438 (51) |  |  |  |  |
| Radiotherapy | 181 (23) | 211 (25) |  |  |  |  |
| Chemoradiation | 164 (21) | 202 (24) |  |  |  |  |
| **Charlson comorbidity index—no(%)** | |  | **<0.001** |  |  | 0.97 |
| Mild (0-2) | 220 (28) | 155 (18) |  | 6 (8) | 13 (8) |  |
| Moderate (3-4) | 299 (38) | 310 (36) |  | 22 (29) | 44 (28) |  |
| Severe (≥5) | 264 (34) | 386 (46) |  | 47 (63) | 102 (64) |  |
