# Supplementary material for: Sex-based survival disparities in patients with stage I–III non-small cell lung cancer vary by treatment modality
Source: Front Oncol. 2026 Sep 18;16:1868017. doi: 10.3389/fonc.2026.1868017 (PMC13630633; doi:10.3389/fonc.2026.1868017)
Supplement: Supplementary file 2 [file Table2.docx]

| **Demographics** | **Surgery cohort (n=876)** |  |  |
| --- | --- | --- | --- |
| **Population** | **Female** | **Male** | **P-value** |
|  | n=438 | n=438 |  |
| **Age at diagnosis—years** |  |  | **<0.001** |
| Mean±SD | 64.7 ± 9.2 | 67.3 ± 9.0 |  |
| Median(range) | 66 (32-84) | 68 (18-88) |  |
| **ECOG PS at diagnosis—no(%)** |  |  | 0.32 |
| 0 | 293 (67) | 279 (64) |  |
| 1 | 120 (27) | 139 (32) |  |
| >1 | 21 (5) | 14 (3) |  |
| Unknown | 4 (1) | 6 (1) |  |
| **Smoking status—no(%)** |  |  | **0.005** |
| Current | 168 (38) | 144 (33) |  |
| Former | 213 (49) | 259 (59) |  |
| Never | 47 (11) | 25 (6) |  |
| Unknown | 10 (2) | 10 (2) |  |
| **Year of diagnosis—no(%)** |  |  | **0.07** |
| ≤2016 | 24 (6) | 45 (10) |  |
| 2017 | 145 (33) | 131 (30) |  |
| 2018 | 136 (31) | 132 (30) |  |
| 2019 | 133 (30) | 130 (30) |  |
| **Type of diagnosis—no(%)** |  |  | 0.65 |
| Pathologically proven | 429 (98) | 427 (97) |  |
| Imaging only | 9 (2) | 11 (3) |  |
| **Histopathology if known—no(%)** |  |  | **<0.001** |
| Adenocarcinoma | 268 (63) | 208 (49) |  |
| Squamous cell carcinoma | 95 (22) | 179 (42) |  |
| NSCLC NOS | 17 (4) | 11 (2) |  |
| Other | 49 (11) | 29 (7) |  |
| **Clinical stage at baseline—no(%)** |  |  | **<0.001** |
| I | 264 (60) | 207 (47) |  |
| II | 114 (26) | 129 (30) |  |
| III | 60 (14) | 102 (23) |  |
| **Charlson comorbidity index—no(%)** | |  | **0.003** |
| Mild (0-2) | 143 (33) | 100 (23) |  |
| Moderate (3-4) | 170 (39) | 181 (41) |  |
| Severe (≥5) | 125 (28) | 157 (36) |  |
