# Supplementary material for: Sex-based survival disparities in patients with stage I–III non-small cell lung cancer vary by treatment modality
Source: Front Oncol. 2026 Sep 18;16:1868017. doi: 10.3389/fonc.2026.1868017 (PMC13630633; doi:10.3389/fonc.2026.1868017)
Supplement: Supplementary file 3 [file Table3.docx]

| **Demographics** | **Radiotherapy cohort (n=392)** |  |  |
| --- | --- | --- | --- |
| **Population** | **Female** | **Male** | **P-value** |
|  | n=181 | n=211 |  |
| **Age at diagnosis—years** |  |  | **0.007** |
| Mean±SD | 72.1 ± 9.0 | 74.5 ± 8.4 |  |
| Median(range) | 73 (49-93) | 76 (45-89) |  |
| **ECOG PS at diagnosis—no(%)** |  |  | 0.92 |
| 0 | 47 (26) | 52 (24) |  |
| 1 | 76 (42) | 92 (44) |  |
| >1 | 47 (26) | 57 (27) |  |
| Unknown | 11 (6) | 10 (5) |  |
| **Smoking status—no(%)** |  |  | **0.002** |
| Current | 82 (45) | 57 (27) |  |
| Former | 85 (47) | 137 (64) |  |
| Never | 3 (2) | 3 (2) |  |
| Unknown | 11 (6) | 14 (7) |  |
| **Year of diagnosis—no(%)** |  |  | 0.36 |
| ≤2016 | 10 (6) | 17 (8) |  |
| 2017 | 48 (26) | 65 (31) |  |
| 2018 | 59 (33) | 70 (33) |  |
| 2019 | 64 (35) | 59 (28) |  |
| **Type of diagnosis—no(%)** |  |  | 0.58 |
| Pathologically proven | 97 (54) | 119 (56) |  |
| Imaging only | 84 (46) | 92 (44) |  |
| **Histopathology if known—no(%)** |  |  | 0.55 |
| Adenocarcinoma | 49 (51) | 53 (45) |  |
| Squamous cell carcinoma | 39 (40) | 48 (40) |  |
| NSCLC NOS | 8 (8) | 13 (11) |  |
| Other | 1 (1) | 5 (4) |  |
| **Clinical stage at baseline—no(%)** |  |  | **0.05** |
| I | 145 (80) | 151 (72) |  |
| II | 26 (14) | 34 (16) |  |
| III | 10 (6) | 26 (12) |  |
| **Radiotherapy regimen—no(%)** | |  | 0.07 |
| Stereoablative 154 (85) | | 168 (80) |  |
| Conventional 21 (12) | | 40 (19) |  |
| Unknown 6 (3) | | 3 (1) |  |
| **Charlson comorbidity index—no(%)** | |  | **0.02** |
| Mild (0-2) | 14 (8) | 9 (4) |  |
| Moderate (3-4) | 58 (32) | 47 (22) |  |
| Severe (≥5) | 109 (60) | 155 (74) |  |
