# Supplementary material for: Sex-based survival disparities in patients with stage I–III non-small cell lung cancer vary by treatment modality
Source: Front Oncol. 2026 Sep 18;16:1868017. doi: 10.3389/fonc.2026.1868017 (PMC13630633; doi:10.3389/fonc.2026.1868017)
Supplement: Supplementary file 4 [file Table4.docx]

| **Demographics** | **Chemoradiation cohort (n=366)** |  |  |
| --- | --- | --- | --- |
| **Population** | **Female** | **Male** | **P-value** |
|  | n=164 | n=202 |  |
| **Age at diagnosis—years** |  |  | **<0.001** |
| Mean±SD | 63.3 ± 9.0 | 68.2 ± 8.8 |  |
| Median(range) | 64 (38-83) | 69 (35-89) |  |
| **ECOG PS at diagnosis—no(%)** |  |  | 0.95 |
| 0 | 86 (52) | 106 (52) |  |
| 1 | 64 (39) | 82 (41) |  |
| >1 | 10 (6) | 10 (5) |  |
| Unknown | 4 (3) | 4 (2) |  |
| **Smoking status—no(%)** |  |  | 0.71 |
| Current | 78 (47) | 91 (45) |  |
| Former | 77 (47) | 103 (51) |  |
| Never | 6 (4) | 4 (2) |  |
| Unknown | 3 (2) | 4 (2) |  |
| **Year of diagnosis—no(%)** |  |  | 0.26 |
| ≤2016 | 12 (8) | 12 (6) |  |
| 2017 | 41 (25) | 59 (29) |  |
| 2018 | 53 (32) | 48 (24) |  |
| 2019 | 58 (35) | 83 (41) |  |
| **Type of diagnosis—no(%)** |  |  | 0.37 |
| Pathologically proven | 164 (100) | 201 (99) |  |
| Imaging only | 0 (0) | 1 (1) |  |
| **Histopathology if known—no(%)** |  |  | **<0.001** |
| Adenocarcinoma | 92 (56) | 70 (35) |  |
| Squamous cell carcinoma | 49 (30) | 96 (48) |  |
| NSCLC NOS | 18 (11) | 32 (16) |  |
| Other | 5 (3) | 3 (1) |  |
| **Clinical stage at baseline—no(%)** |  |  | 0.29 |
| I | 0 (0) | 3 (1) |  |
| II | 6 (4) | 8 (4) |  |
| III | 158 (96) | 191 (95) |  |
| **Adjuvant durvalumab—no(%)** | |  | 0.42 |
| Yes 56 (34) | | 61 (30) |  |
| No 108 (66) | | 141 (70) |  |
| **Charlson comorbidity index—no(%)** | |  | **0.02** |
| Mild (0-2) | 63 (39) | 46 (23) |  |
| Moderate (3-4) | 71 (43) | 82 (40) |  |
| Severe (≥5) | 30 (18) | 74 (37) |  |
